# Supplementary material for: Development and validation of the AUDEXCEL algorithm as a diagnostic tool for occupational noise-related hearing disorder
Source: PeerJ. 2025 Oct 10;13:e20149. doi: 10.7717/peerj.20149 (PMC12517279; doi:10.7717/peerj.20149)
Supplement: Supplemental Information 3 [file peerj-13-20149-s003.docx]

**AUDEXCEL User Manual**

Step 1: Key in the audiometric readings for each individual, in the order of baseline, annual, and repeated (for STS).


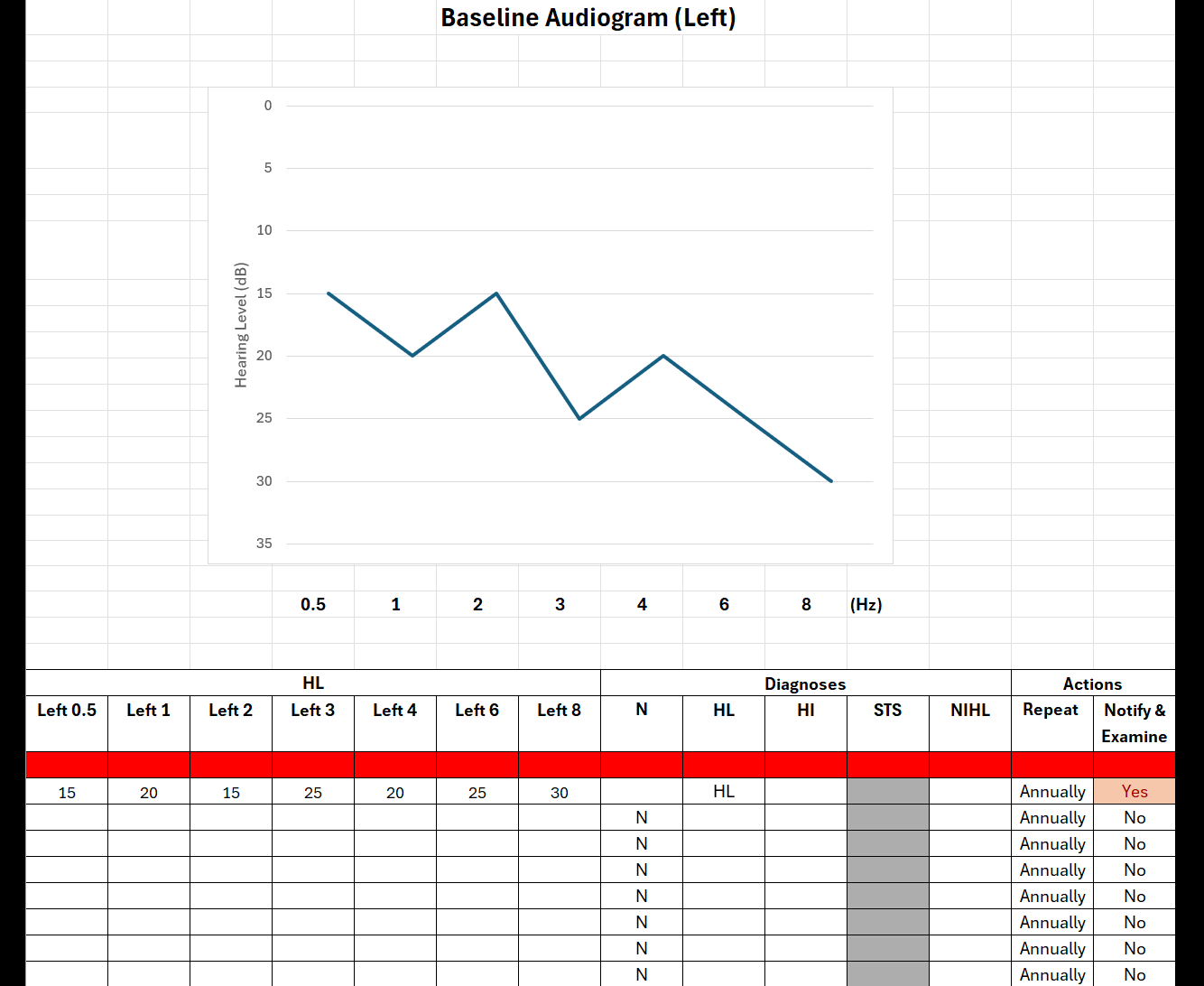


Step 2: The diagnoses for both left and right ears will be automatically generated.


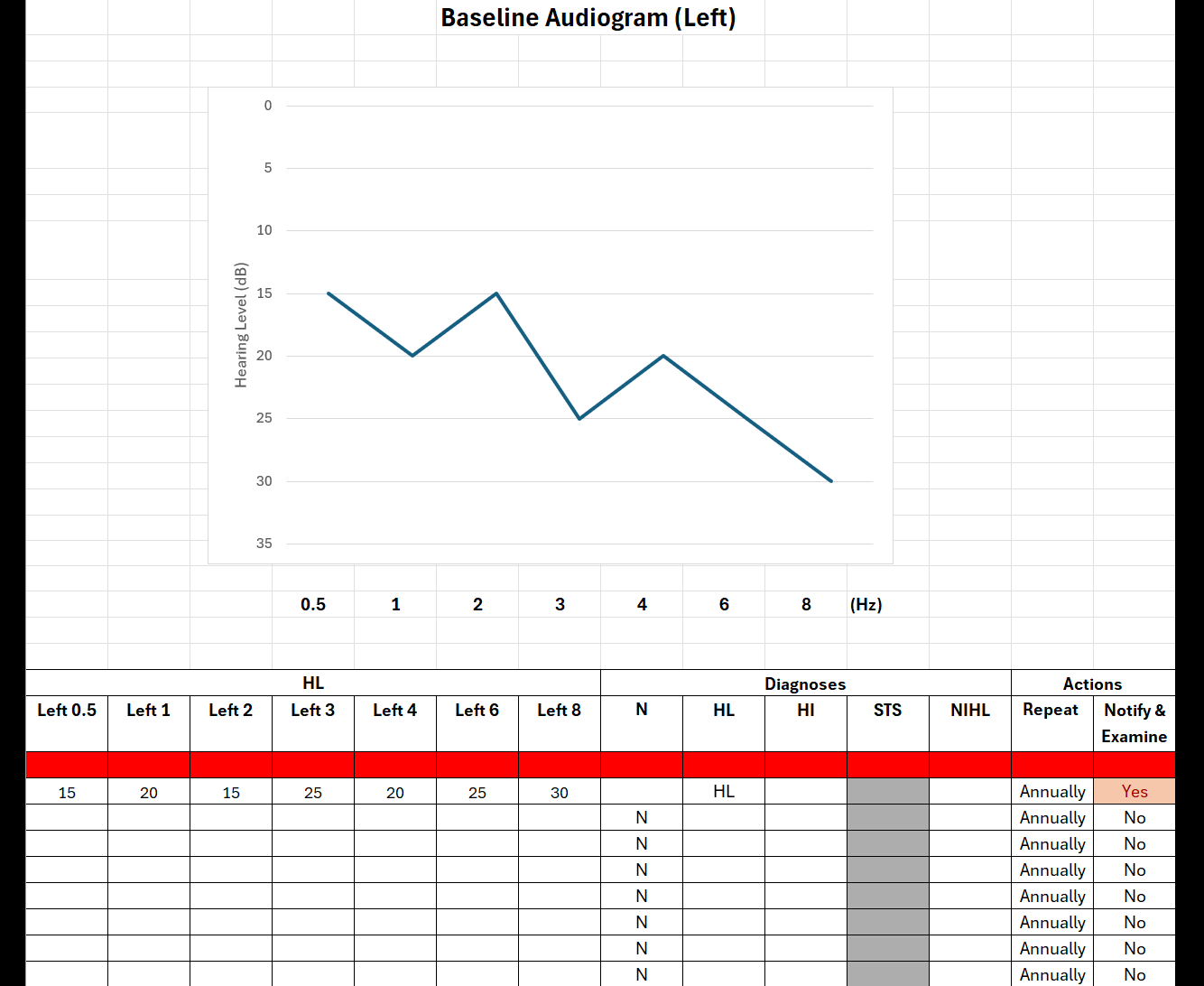


Step 3: Please take note of the actions required to take for each individual (e.g., to repeat audiometry within 3 months, repeat audiometry annually and/or to notify the authority).


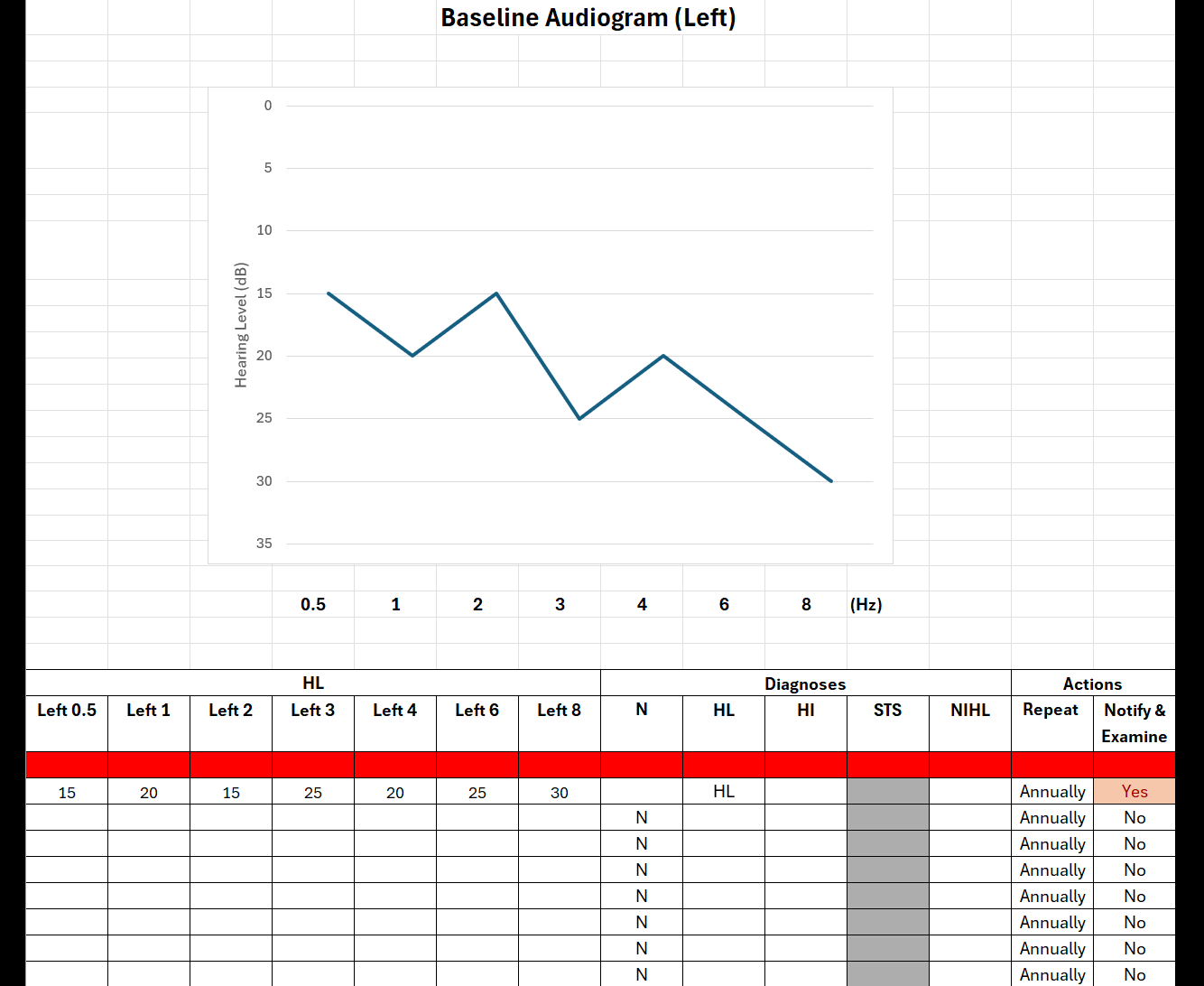


Step 4: To visualise the audiogram for each individual, click the line plot of the audiogram and move the blue bar to highlight the audiometric readings of interest.


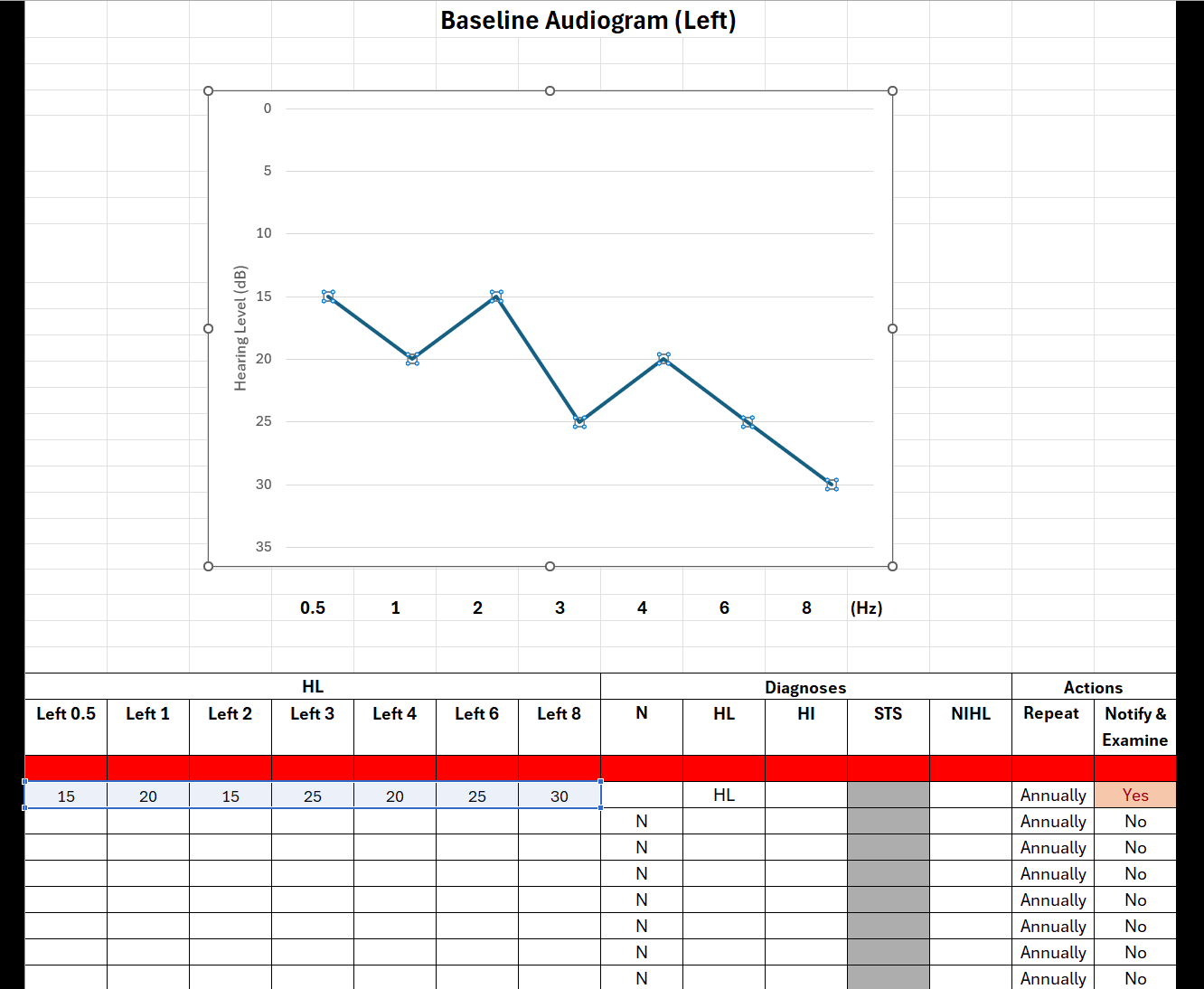


Line Plot

**Step 5: Key in the audiometric readings for another individual at the next line.**


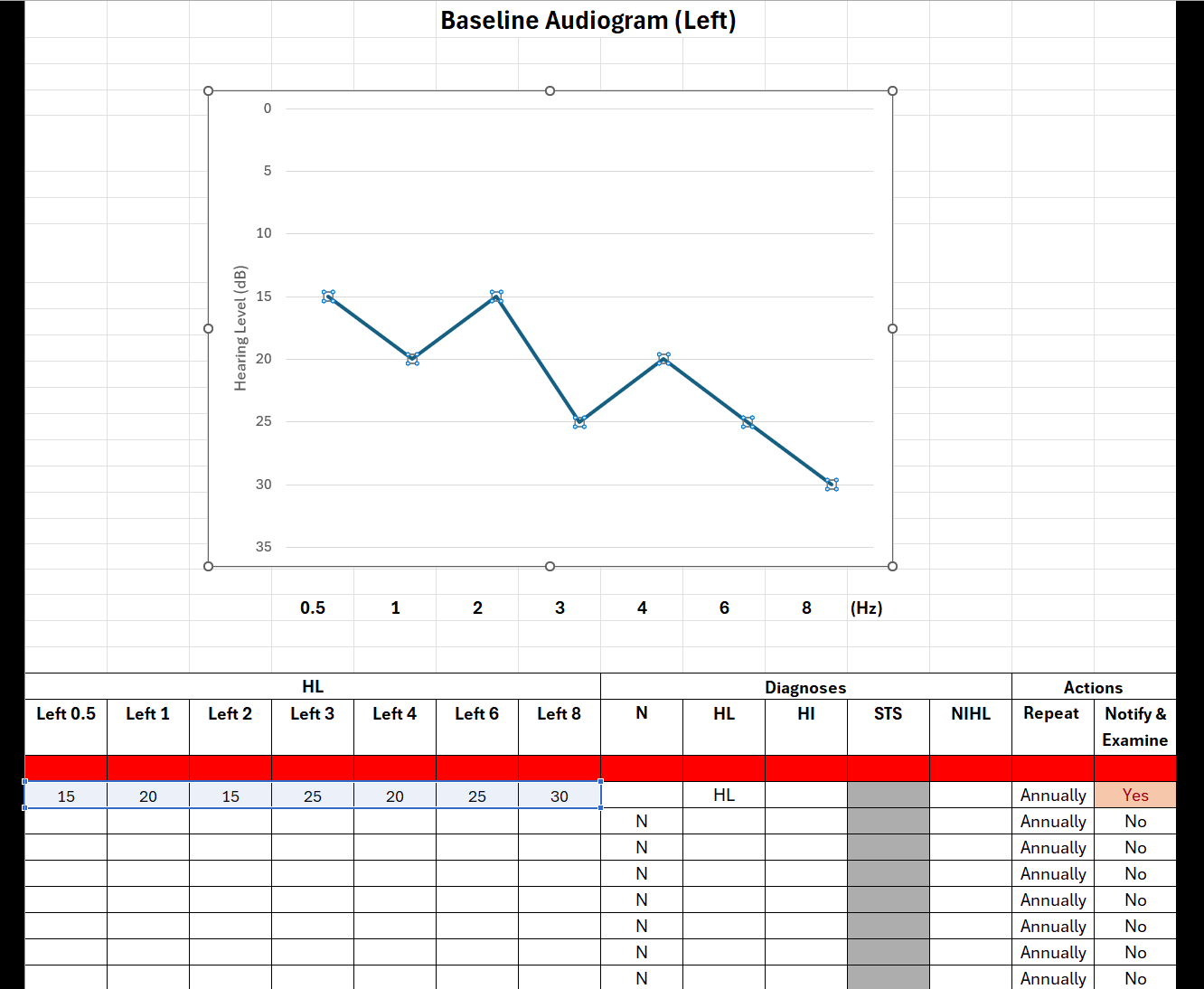


Next individual
